# Ethical Dilemma: Is it Worthwhile Operating an End-Stage Pancreatic Cancer Patient with Acute Mesenteric Artery Ischemia?

**DOI:** 10.15388/Amed.2021.28.2.17

**Published:** 2021-12-17

**Authors:** Christos Damaskos, Nikolaos Garmpis, Anna Garmpi, Vasiliki E. Georgakopoulou, Alexandros Patsouras, Georgia Sypsa, Athanasios Syllaios, Efstathios A. Antoniou

**Affiliations:** Renal Transplantation Unit, Laiko General Hospital, Athens, Greece; N.S. Christeas Laboratory of Experimental Surgery and Surgical Research, Medical School, National and Kapodistrian University of Athens, Athens, Greece; N.S. Christeas Laboratory of Experimental Surgery and Surgical Research, Medical School, National and Kapodistrian University of Athens, Athens, Greece; Second Department of Propedeutic Surgery, Laiko General Hospital, Medical School, National and Kapodistrian University of Athens, Athens, Greece; First Department of Propedeutic Internal Medicine, Laiko General Hospital, Medical School, National and Kapodistrian University of Athens, Athens, Greece; Department of Pulmonology, Laiko General Hospital, Athens, Greece; First Department of Pulmonology, Sismanogleio Hospital, Athens, Greece; N.S. Christeas Laboratory of Experimental Surgery and Surgical Research, Medical School, National and Kapodistrian University of Athens, Athens, Greece; Department of Radiology, Laiko General Hospital, Athens, Greece; School, National and Kapodistrian University of Athens, Greece; First Department of Surgery, Laiko General Hospital, Medical School, National and Kapodistrian University of Athens, Athens, Greece; N.S. Christeas Laboratory of Experimental Surgery and Surgical Research, Medical School, National and Kapodistrian University of Athens, Athens, Greece; Second Department of Propedeutic Surgery, Laiko General Hospital, Medical School, National and Kapodistrian University of Athens, Athens, Greece

**Keywords:** End-stage pancreatic cancer, Acute mesenteric artery ischemia, Surgery, Treatment, Ethical dilemma

## Abstract

Pancreatic cancer is as an aggressive malignancy with low survival rates. We present the first case of an operation of acute mesenteric ischemia performed in a patient with end-stage pancreatic adenocarcinoma. Through this case, we also discuss raising concerns regarding the management of severe complications such as acute mesenteric ischemia in patients with progressed pancreatic carcinoma. How ethical is to leave patients untreated? The decisions for management of patients with advanced disease are strongly based on the expected quality of life, ethical principles, different religions and spiritualities, and the burden of healthcare cost.

## Abstract (lt)

**Moralinė dilema – ar verta operuoti paskutinės stadijos kasos vėžiu sergantį pacientą, kuriam pasireiškė ūminė mezenterinė išemija?:**

**Santrauka. **Kasos vėžys yra agresyvus piktybinis darinys, jam pasireiškus tikimybė išgyventi yra maža. Aprašome pirmąjį atvejį, kai paskutinės stadijos kasos adenokarcinoma sergančiam pacientui atlikta ūminės mezenterinės išemijos operacija. Aptardami šį atvejį, taip pat aptarsime susirūpinimą keliančių sunkių komplikacijų, tokių kaip ūminės mezenterinės išemijos valdymas pacientams, sergantiems progresavusia kasos karcinoma. Ar moralu negydyti pacientų? Sprendimai, kaip gydyti pacientus, kurių liga yra pažengusi, priklauso nuo tikėtinos gyvenimo kokybės, moralės principų, skirtingų religijų ir dvasingumo bei sveikatos priežiūros paslaugų kainos.

**Raktažodžiai:** paskutinės stadijos kasos vėžys, ūminė mezenterinė išemija, operacija, gydymas, moralinė dilema

## Introduction

Pancreatic cancer is regarded as an aggressive malignancy with a five-year survival rate of 8% [1]. Its treatment options are mostly radiotherapy and palliative care. Surgery cannot usually be performed at the time of diagnosis [2]. The chemotherapy regimens demonstrate controversial efficacy [3]. Complications such as mesenteric thrombosis, mainly venous and less frequently arterial thrombosis, have been reported in the literature [4]. Patients older than 60 years old, comorbidities, colon involvement, bowel resection and duration of symptoms of acute mesenteric ischemia are considered as negative prognostic factors [5].

## Case Report

On the occasion of a case of an 82-year-old male patient, with known history of diabetes mellitus and hypertension, who presented to our emergency department due to acute abdominal pain, we discuss the ethical dilemma of operating an end-stage pancreatic cancer patient with acute mesenteric artery ischemia or not. The patient presented with the following vital signs: 103 pulses/min, respiratory rate 22/min, blood pressure 112/52 mmHg and temperature of 36.8 ^o^C. Even though we could establish communication with the patient, he was not oriented in space and time. The patient was accompanied by his wife who was fully informed prior to the surgery about all the causes that led us to this decision. 

**Figure 1:**
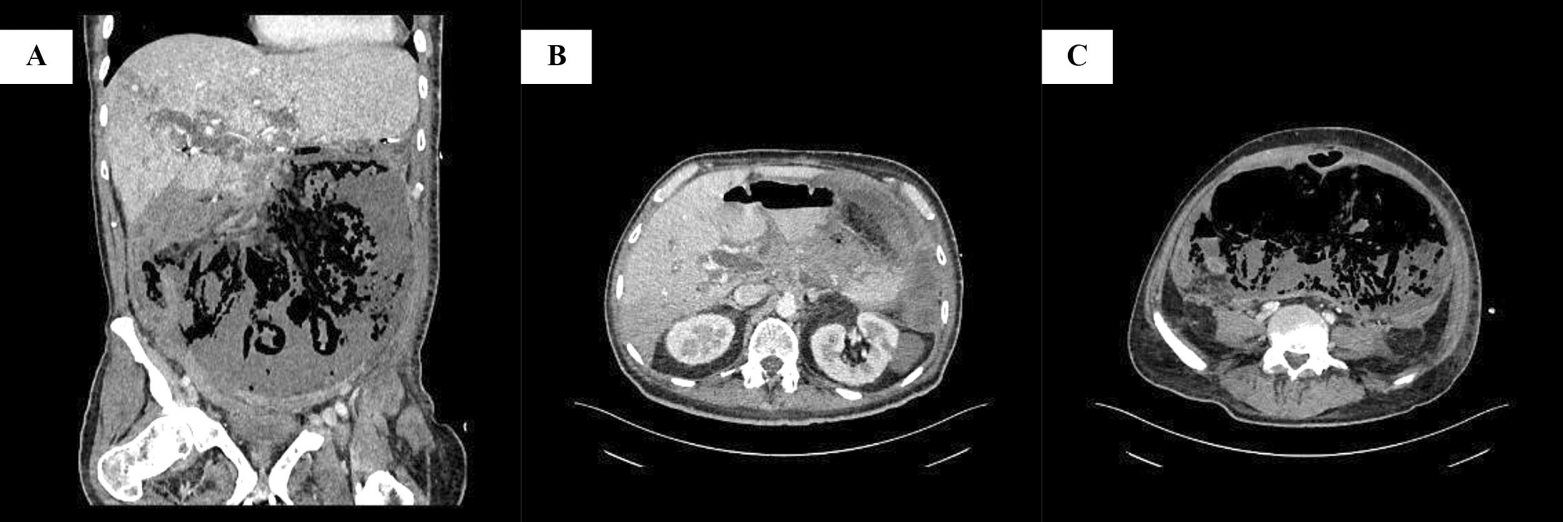
Preoperative computed tomography findings. **A:** Coronal view. Large fluid and air collection inside the peritoneal cavity, surrounded by thickened peritoneum with mild enhancement, due to peritonitis following bowel perforation; diffuse bowel wall thinning with intramural gas bubbles, suggestive of mesenteric ischemia; periportal edema and small peripheral area of hypoattenuation in the liver. **B:** Transverse view at the level of superior mesenteric artery origin. Extensive hypoattenuating mass located predominantly on the pancreatic body and less on the head, with invasion of the superior mesenteric artery from the level of its origin from the aorta; complete absence of attenuation of the superior mesenteric artery due to occlusion; the lesion extends to the hepatoduodenal ligament and porta hepatis; and dilatation of common bile duct. **C: **Transverse view at the level of L5. Large collection of fluid and gas in the peritoneal cavity, with thickened peritoneum; and thinning and absence of definition of the bowel wall, due to acute mesenteric ischemia.

The abdominal clinical examination revealed diffuse tenderness, decreased bowel sounds and loss of dumbness subdiaphragmatically during percussion. These findings suggested bowel perforation as a possible diagnosis. The laboratory tests revealed metabolic acidosis, lactate, elevated d-dimers and leukocytosis. A computed tomography (CT) with intravenous contrast media was carried outwhich demonstrated air subdiaphragmatically and occlusion of the superior mesenteric artery. Other findings were a 5x6 cm lesion in the body of the pancreas with concomitant peritoneal metastasis, suggesting Stage IV pancreatic adenocarcinoma (Figure 1).

A resuscitation effort with fluids and antibiotics of broad spectrum for anaerobic bacteria took place. Vasoconstrictive drugs were not administrated in order to avoid further bowel ischemia. Due to the diffuse peritonitis and the concern for the bowel viability, conservative treatment with fibrinolytics for the destruction of the thrombus was abandoned, and an emergency laparotomy was the choice of treatment. Despite the revascularization, the small bowel was necrotic, and its total resection followed. A gastrostomy and a duodenostomy were performed. The patient was admitted to the intensive care unit (ICU) extubated, where he received prompt treatment with multi-organ support including fluid resuscitation and nutriments via the feeding tube. Unfortunately, the patient suffered from sepsis and respiratory failure. He was once again intubated, and died in the ICU after 30 days. 

## Discussion

As presented, this case had an emergency nature. Due to extensive peritonitis the surgery was the only way to go. The only dilemma could have been arisen during the surgery was either to perform small bowel resection, gastrostomy and duodenostomy or do nothing and close the patient up, supporting him afterwards until the inevitable.

This case is the first to report an operation of acute mesenteric ischemia performed in a patient with end-stage pancreatic adenocarcinoma. Furthermore, the case raises concerns regarding the management of severe complications such as acute mesenteric ischemia in patients with progressed pancreatic carcinoma. Is this aggressive treatment worthwhile in a patient with such a low long-term survival and comorbidities? Was any beneficial impact on the patient after the operation? What would be the quality of life of the patient? The lack of beds in the ICU unit and the choice of patients entering are important issues which needs to be mentioned. On the other hand, is it ethical to leave a patient untreated and in pain?

The management of patients with advanced disease is strongly influenced by their expected quality of life, by ethical principles, different religions and spiritualities, and the by the burden of healthcare cost. For appropriate decision making, extensive patient information and understanding of the treatment choices, including the potential benefits and harms, are essential. The primary scope of cancer treatment has always been to increase survival. However, quality of life is an increasingly recognized important issue. [6] According to some studies, older patients prefer quality of life, a finding that is not surprising, taking into account natural limitations to life expectancy and the reduced quality of life related to advanced age [7]. On the other hand, younger cancer patients prefer tolerating aggressive treatments in order to increase survival [8].

One of the dilemmas that can occur, regards the performance or not of medical interventions in end-stage cancer patients. These interventions range from minor, such as medication administration, to major, such as mechanical ventilation or surgery. The decision for avoiding these interventions is usually based on the fact that the burdens outweigh the benefits received. Life-sustaining therapies may sometimes lead to prolonged suffering, decreasing the patient’s quality of life. Advance directives have great importance. Advance directives are documents that enable patients to make their decisions about provided medical care known to their family and health professionals, in case that they are unable to make those decisions themselves, helping prevent the initiation of some life sustaining treatments and reduce overall costs of worthless medical care. [9]

Perspectives about management of an end-stage cancer patient vary among different religions, with nations’ culture having significant impact on beliefs and practices regarding termination of care, artificial nutrition and hydration, pain relief and autopsy. Catholics in Europe have been found to prefer withdrawing treatment in an end-of-life situation as opposed to Protestants, while American Roman Catholics are three times more likely to refuse the withdrawal of life support than Protestants. The Judeo-Christian opinion endorses the administration of pain medications as long as the aim is to provide comfort to the dying patient. On the contrary, Eastern religions like Hinduism and Buddhism often do not agree with the use of opioids at the end of life due to the undesirable decreased level of consciousness at the time of death. [10] According to Islam, treatment for cancer must be continued until it is determined that the disease is undoubtedly terminal [11] and it is necessary to provide everything the patient needs for normal sustenance [12].

The cost to society of providing medical care to people with cancer at the end of life is significant [13]. The high cost of medical care at the end of life is a past development, since a study in 1961 study demonstrated that hospital and other institutional expenses of sick adults who died were approximately three times higher of the expenses of sick adults who survived. These costs raise the question if resources are being wasted on the end-stage cancer patients (especially on elderly ones) or if they should be more productively allocated to other patients. [14] It has been reported that hospice care is effective in saving a large amount of these medical costs, especially when patients are referred to this type of care earlier [15].

Taking all the above into account and in order to help making the right decisions, the Committee on Bioethics (DH-BIO) of the Council of Europe has developed a guide based on the principles adopted by the Convention on Human Rights and Biomedicine (Oviedo Convention, ETS No. 164, 1997). [16] The law in Greece does not specify any guidelines regarding these cases. It only states our obligation as physicians to do whatever possible to maintain and prolong any patients’ life, regardless its quality. Due to the emergency of this case no particular discussion among physicians took place. Surgery is, without any doubt, the treatment of choice in patients with perforated small bowel, free air in the peritoneum and peritonitis. From the standpoint of the authors, we have the impression that the correct decision was made because we prolonged the patients’ life. The patient felt no pain or hunger until his final moments.

## Conclusions

In complex life-related situations, the primary purpose of any medical treatment is to alleviate and focus on quality of life. On the other hand, clinical experience shows that, at the end of their life, patients may be vulnerable and have difficulty or not be able to express an opinion. We should not forget, however, that the main thread of any discussion of medical treatment decisions must be the respect for the dignity of the individual. So, in all these situations where the clinician has ethical dilemmas, decision making should be based on medical procedures taking into account the aforementioned considerations.
